# The association between health literacy and pesticide use behaviors among sweet corn farmers in the Pak Chong district of Thailand: a cross-sectional study

**DOI:** 10.12688/f1000research.18398.2

**Published:** 2019-05-15

**Authors:** Theerachai Pobhirun, Somdej Pinitsoontorn

**Affiliations:** 1 Pakchong Nana Hospital, 400 Mittraphap Road, Tambon Pak Chong, Amphoe Pak Chong, Nakhon Ratchasima, 30130, Thailand; 2 Department of Community Medicine, Faculty of Medicine, Khon Kaen University, 123 Mittraphap Road, Mueang District, Khon Kaen, 40002, Thailand

**Keywords:** Pesticides, Health literacy, Sweet corn, Harm reduction, Farmers, Pattern, Behavior

## Abstract

**Background**: Pesticide toxicity is an important health problem in Thailand due to the intensive use of hazardous pesticides.  This study aimed to determine and discuss patterns of pesticide use, health literacy, pesticide use behaviors and whether there is an association between health literacy and pesticide use behaviors among sweet corn farmers in the Pak Chong district, Thailand.

**Methods**: This work was carried out between May 2017-July 2017 and 161 participants were enrolled. Participant questionnaires were completed during face-to-face interviews.

**Results**:  This study found the response rate was 98.98%. 161 farmers were interviewed about patterns of chemical pesticide use. Two of the pesticides used in the pre-planting phase were moderately toxic: paraquat (used by 55.2% of farmers) and imidacloprid (used by 15.5% of farmers). In the pre-emergence phase, participants reported using two moderately toxic pesticides: alachlor (used by 48.8% of farmers) and chlorpyrifos (used by 2.4% of farmers). At the post-emergence phase, participants reported using six moderately toxic pesticides: chlorpyrifos (used by 60.7% of farmers), paraquat (used by 38.1% of farmers), imidacloprid (used by 7.2% of farmers), 2-4D (used by 3.6% of farmers), abamectin (used by 3.6% of farmers) and cypermethrin (used by 1.2% of farmers). Health literacy levels were moderate level (Mean score = 91.62, SD = ± 7.06) and pesticide use behaviors were low level (Mean score = 67.80, SD = ± 4.04). When examining the association between health literacy and pesticide use behaviors, we found that functional literacy was significantly associated with pesticide use behaviors. This suggests that health literacy, which includes self-management and decision-making skills, should be given greater attention as pesticide use behaviors were unsafe.

**Conclusion**: It may be necessary to develop approaches to reduce pesticide use and promote health literacy, thereby protecting farmers, consumers, the environment (soil, water, and air) and ecosystems from pesticide-related hazards.

## Introduction

Agricultural production accounts for 10% of the Kingdom of Thailand’s gross domestic product (GDP) and 60% of employment. As in many developing countries, in order to achieve high yields, farmers in Thailand use pesticides and herbicides to control pests, weeds and other pathogens. Despite following stricter regulations, increasing evidence of the health risks associated with exposure to pesticides and pesticide residues contaminating food, water and air ^1, 2^, imports of insecticides, herbicides, and fungicides have peaked in the last decade, particularly between 2011 to 2017 ^3^. The World Health Organization (WHO) classification of pesticides by hazard has been aligned with the GHS (The Globally Harmonized System of Classification and Labelling of Chemicals) acute toxicity hazard categories: Ia = extremely hazardous; Ib = highly hazardous; II = moderately hazardous; III = slightly hazardous; U = unlikely to present an acute hazard; O = Obsolete as pesticide (not classified) ^3^. Those pesticides classified as toxic include glyphosate (III), paraquat (II), 2,4-D-dimethylammonium (II), atrazine (III), ametryn (II), 2,4-D-sodium, diuron (III), propyl (O), chlorpyrifos (II), and mancozeb (U). Currently, the Thai Pesticide Alert Network (Thai-PAN) is campaigning and working with the government of Thailand to ban four pesticides (paraquat, glyphosate, chlorpyrifos, and 2,4D) due to their long-term health effects on humans. In addition, Thailand has adopted the Sufficiency Economy Philosophy as a guideline for agriculture. According to this philosophy, building immunity to environmental changes prompts individuals and their communities to be aware of the impacts their actions may have on the environment and, subsequently, their livelihoods, an awareness which leads them to live in harmony with nature ^4^. The environmental aspects of this guideline enable people and communities to realize the effects of pesticides on health, environment and livelihoods.

However, pesticide use trends indicate that pesticide use is increasing ^5^. Although pesticides are beneficial for the control of pests, there are serious concerns for humans and animals regarding associated health risks. Pesticides are chemicals which have been designed to be toxic to pests and, in many cases, their toxic nature can also be harmful to human health ^1, 6, 7^ and cause environmental pollution ^8^. There have been reports of strong associations between exposure to pesticides and cancer ^9^, and studies have shown that the exposure of pregnant women and breastfeeding mothers to pesticides is a risk factor for low birth weight, congenital birth defects, growth defects, learning disabilities and miscarriage ^1, 10^. Previous evidence suggests that farmers have awareness and a basic understanding of insecticides, pesticides and various other chemicals used in farming practices ^11– 13^ However, this awareness and knowledge alone might not lead to reduced use of the various chemicals when farming. Health literacy (HL) is a relatively new concept for social and medical sciences research. HL is the ability of individuals to understand basic health information and gain access to essential services, thereby enabling them to make informed decisions ^8, 9^. Health literacy can be separated into three levels ^14– 16^: (1) Functional literacy, which focuses on the ability to read, understand and access pesticide information; (2) Interactive literacy, which involves the use of cognitive skills and operates in a social environment that supports social participation in health-related issues in the community; (3) Critical literacy, which is the ability to evaluate health issues and determine the challenges of these issues.

The patterns of pesticide use were studied in three phases of sweet corn farming: pre-planting, pre-emergence and post-emergence. Sweet corn farmers use various methods when applying pesticide such as mixing, loading, spraying and washing equipment. Mixing involves weighing or measuring the pesticide in some fashion and mixing the measured concentrated product with a diluent. Loading involves pouring the diluted pesticide into the spray equipment. Spraying involves the application of the pesticide to control pests by spraying. This is the most common activity for farmers. Typical equipment used may be backpack sprayers or hand-held tank sprayers. Washing involves cleaning the equipment used for pesticide application which may be contaminated during the spraying operation. Pesticide use patterns included the number, type, toxicity and concentration of pesticides used and pesticide use behaviors. There are very few studies about pesticide use among sweet corn farmers. The aim of this research was to determine and discuss patterns of pesticide use, HL of pesticides used, pesticide use behaviors and the association between HL and the behaviors of sweet corn farmers.

## Methods

This research applied a cross-sectional, quantitative design to identify determinants of health literacy and pesticide use. This study was carried out from May 2017 to July 2017. The participants were sweet corn farmers in the Pak Chong district of Nakhon Ratchasima province, Thailand. Specifically, the study was conducted in the Klangdong, Chanthuek, Wang Sai and Mu Si sub-districts of the Pak Chong district as these are the regions in which sweet corn is grown. Within these regions, 194 sweet corn farmers were identified through visits to the farms in the area (purposive sampling). However, 33 farmers were unwilling to be interviewed.

The eligibility criteria of the respondents include sweet corn farmers who live in Pak Chong district, who had used a pesticide for at least 6 months, who could read and write and who were willing to participate in the study. Written informed consent was obtained from all the participants (n = 161). Interviews were conducted by the author (T.P.) using a questionnaire, which included questions about socio-demographic factors and patterns of pesticide use, and answers from farmers were given orally. In order to assess health literacy, the three levels were further separated into 6 dimensions: cognitive skills, access, communication skills, self-management, media literacy and decision-making skills. Cognitive skills refers to the knowledge and understanding the participant has about the correct use of pesticides. Access refers to the access the participant has to pesticide effect information and health services. Communication skills refers to the ability of the participant to listen, speak, read and write about pesticide use. Self-management refers to the ability of the participant to protect their health and reduce the impact on the environment of pesticide application. Media literacy refers to the ability to compare information regarding pesticide efficacy from media such as newspapers, radio, television and pesticide advertisements. Decision-making skills refers to the ability of the participant to decide what pesticides to use and how to use them in a way that does not have a negative impact on their health or the environment.

Three experts from Thai-PAN (Thai Pesticide Alert Network) verified the content validity. The consistency index for all items was 0.61. A pilot test (sample size of 30) was used to achieve clarity for the questionnaires. Cronbach’s alpha, a measure of internal consistency, was 0.72 for the HL of pesticide use section of the questionnaire and 0.77 for the pesticide use behaviors section. Responses to questions 1–10 (cognitive skills) were marked as correct or incorrect. Responses to the remaining questions were scored qualitatively, using a 5-point Likert scale. The item responses ranged from 1 (very low) to 5 (very high). The HL of pesticide use section was composed of 36 items and it was separated into 6 parts (access, cognitive skills, communication skills, self-management, media literacy and decision-making skills). Responses to the 36 items were scored as low, moderate or high levels of health literacy for pesticide use. The maximum score for health literacy was 135. A score of <81 was considered to be low, a score of 81–108 was considered to be moderate and a score of >107 was considered to be high. A breakdown of the score thresholds for each dimension and level of health literacy can be found in Table 1. The pesticide use behaviors section was composed of 20 items, divided into 3 parts (before application, pesticide application and after application). Responses to the 20 items were scored as low, moderate and high standards of pesticide use behaviors. The maximum score for pesticide use behaviors was 100. A score of <67 was considered to be low, a score of 67–69 was considered to be moderate and a score of >69 was considered to be high. A breakdown of the score thresholds for each phase of pesticide application can be found in Table 2.

**Table 1.** Score thresholds for the health literacy section of the questionnaire.

HL Dimension	Max Score	Low	Moderate	High
Overall	135	<81	81-107	>107
Cognitive	10	<6	6-7	>7
Access	30	<18	18-23	>23
Communication	25	<15	15-19	>19
Self-management	25	<15	15-19	>19
Media literacy	25	<15	15-19	>19
Decision-making	20	<12	12-15	>15
Functional HL	35	<21	21-27	>27
Interactive HL	55	<33	33-43	>43
Critical HL	45	<27	27-35	>35

**Table 2.** Score thresholds for the pesticide use behaviors section of the questionnaire.

Application phase	Max Score	Low	Moderate	High
Overall	100	<67	67-69	>69
Before	20	<14	14-15	>15
Application	55	<33	33-34	>34
After	25	<20	20	>20

Descriptive statistics, such as frequencies, percentage, mean and standard deviation (S.D.) are used to report the socio-demographic factors, number of years using pesticides, number of annual pesticide applications, total types of pesticide used in pre-planting, pre-emergence and post-emergence phases of sweet corn farming. In addition, pesticide use behaviors such as; the type of pesticides, the level of toxicity of pesticides and the concentration of pesticides are reported using descriptive statistics. The Chi-squared test was used to test the association between HL and pesticide use behaviors using SPSS (version 20). This analysis could also be performed using a non-proprietary software such as R.

### Ethics statement

During the data collection stage, we had participants provide their written consent. Personal identifiers (names, full addresses) were stripped from the dataset. This research project was approved by the Human Research Ethical Committee of Khon Kaen University (HE601107) based on the principles of the Declaration of Helsinki and the Good Clinical Practice standards of the International Council for Harmonisation of Technical Requirements for Pharmaceuticals for Human Use (ICH).

## Results

### The demographic characteristics of the 161 sweet corn farmers

The majority (54%) of sweet corn farmers were males and between 41–60-years-old (mean 43.14 ± 9.97 years). The average farm size for sweet corn planting was 4.67 acres (min = 1.18, max = 15.81 acres). Most of the participants have been using pesticides for between 6 and 10 years (mean 9.04 ± 4.61 years). Most (64.3%) farmers reported planting two sweet corn crops per year. The two pesticide application methods reported were spraying (reported by 88.8% of farmers) and mixing (reported by 83.2% of farmers).

### Patterns of pesticide use

Patterns of pesticide use were studied over three phases of sweet corn farming. First, the soil is prepared for planting (pre-planting phase). 52.2% of farmers reported spraying paraquat to control broadleaf weeds and 16.7% of farmers reported following this with glyphosate, also to control weeds. Paraquat is a class II toxin and glyphosate is a class III toxin. According to the WHO standard and International Agency for Research on Cancer (IARC), glyphosate is likely carcinogenic to humans. For protection against insect pests, 15.5% of farmers mixed corn seeds with imidacloprid before planting, which is also a class II toxin according to the WHO. In addition, 3.6% of farmers used atrazine to control broadleaf weeds in this phase. In summary, four types of pesticides were used during the pre-planning phase, of which two were considered to be class II toxins.

During the pre-emergence phase, three pesticide types were used: alachlor spray (used by 48.8% of farmers) to control broadleaf weeds, chlorpyrifos spray (used by 2.4% of farmers) to eliminate some pests including insects and worms and atrazine (used by 38.1% of farmers) to control broadleaf weeds. Both alachlor and chlorpyrifos are toxic (class II) according to the WHO. In summary, during the pre-emergence phase, two types of pesticides were class II.

 During the post-emergence phase, pesticides and herbicides are used to eliminate insect pests and weeds. Chlorpyrifos was used by 60.7% of farmers, imidacloprid by 7.2% of farmers, abamectin by 3.6% of farmers and cypermethrin by 1.2% of farmers to eliminate insect pests, all of which are class II toxins. Paraquat (used by 38.1% of farmers) and 2,4D (used by 3.6% of farmers) were used to eliminate weeds, both of which are class II toxins. Atrazine and glyphosate (both used by 2.4% of farmers) belong to toxicity class III ( Table 3). The International Agency for Research on Cancer (IARC) has defined glyphosate and 2,4D (but not atrazine) as carcinogenic.

**Table 3.** The percentage of farmers (n=161) using different pesticides during each of the three stages of sweet corn farming and the toxicity and carcinogenicity to humans of these pesticides.

Type of pesticide	Common name	Stage of sweet corn farming			WHO recommends classification	IARC Carcinogenic to humans
		Pre planting	Pre emergence	Post emergence		
Herbicide	Paraquat	55.2%		38.1%	II	Probably not carcinogenic to humans
	Glyphosate	16.7%		2.4%	III	Probably carcinogenic to humans
	2,4 D			3.6%	II	Possibly carcinogenic to humans
	Atrazine	3.6%	38.1%	2.4%	III	Not classified as carcinogenic to humans
	Alachlor		48.8%		II	Probably not carcinogenic to humans
Pesticide	Abamectin			3.6%	II	Probably not carcinogenic to humans
	Imidacloprid	15.5%		7.2%	II	Probably not carcinogenic to humans
	Chlorpyrifos		2.4%	60.7%	II	Probably not carcinogenic to humans
	Cypermethrin			1.2%	II	Probably not carcinogenic to humans

### Health literacy (HL) of pesticide use

We assessed the sweet corn farmers’ level of HL regarding pesticide use. The majority (91.30%) of the sweet corn farmers enrolled in this study had a moderate level of HL ( Table 4). We found that the cognitive score was mostly (54.66% of farmers) low, access to information was mostly (77.64% of farmers) moderate, communication skills were mostly (96.89% of farmers) low, self-management was mostly (95.65% of farmers) high, media literacy was mostly (74.53% of farmers) low and decision-making skills were moderate for 42.86% of participants and low for 41.61%. When HL was divided into the three levels, functional HL for the majority of participants (53.42%) was moderate and was classed as high for 45.34%. Interactive HL was low for 9.94% and moderate for 87.58% of participants. Critical HL was low for 53.42% and moderate for 45.34% of participants. The overall analysis of pesticide use behaviors found that 37.27 % of farmers had a low standard of pesticide use behaviors and 31.68% of farmers had a moderate standard of pesticide use behaviors. When we analyzed the three phases of pesticide use (1. before pesticide application, 2. pesticide application, 3. after pesticide application), we found that 44.10% of sweet corn farmers had a moderate standard of pesticide use behaviors at the ‘before pesticide application’ phase (mean score 14.2, ± 1.51), 42.24% had a low standard of pesticide use behaviors at the ‘application’ phase (mean score 33.16, ± 2.71) and 43.48% of farmers had a moderate standard of pesticide use behaviors at the ‘after application’ phase (mean score 19.91, ± 1.22) ( Table 5).

**Table 4.** The percentage Thai sweet corn farmers with low, moderate or high levels of health literacy (n=161).

Dimension	Level of HL			Mean Score	S.D.
	Low	Moderate	High		
1. Cognitive skills	54.66%	34.78%	10.56%	6.46	1.28
2. Access	19.88%	77.64%	2.48%	20.38	2.29
3. Communication skills	96.89%	1.86%	1.24%	14.64	2.30
4. Self-management	0%	4.35%	95.65%	22.71	1.31
5. Media literacy	74.53%	21.74%	3.73%	14.30	2.67
6. Decision-making skills	41.61%	42.86%	15.53%	13.12	2.35
**Overall HL**	**6.21**%	**91.30**%	**2.48**%	**91.62**	**7.06**
**Classification**					
Functional literacy	1.24%	53.42%	45.34%	26.84	2.76
Interactive literacy	9.94%	87.58%	2.48%	37.35	2.97
Critical literacy	53.42%	45.34%	1.24%	27.43	3.49

**Table 5.** The percentage of Thai sweet corn farmers with low, moderate or high level of pesticide use behaviors (n=161).

Pesticide use phase	Standard of pesticide use behaviors			Mean Score	S.D.
	Low	Moderate	High		
Before application of pesticide	26.09%	44.10%	29.81%	14.72	1.51
Application pesticide	42.24%	36.02%	21.74%	33.16	2.71
After application of pesticide	32.30%	43.48%	24.22%	19.91	1.22
**Overall**	**37.27**%	**31.68**%	**31.06**%	**67.80**	**4.04**

### The association between health literacy and pesticide use behaviors

The results of the data analysis showed that there was a significant statistical association between health literacy level and pesticide use behaviors (p < 0.05). Of the sweet corn farmers who had a moderate overall health literacy level, 33.33% were inclined towards a high standard of pesticide use behaviors. We found that there was an association between the self-management and decision-making skills dimensions of health literacy and pesticide use behaviors (p < 0.01). Of the sweet corn farmers who had a moderate level of self-management and a high level of decision-making skills, 85.71% and 48.00% were inclined to high standard of pesticide use behaviors, respectively. Regarding functional literacy, there was a significant statistical association with pesticide use behaviors (p < 0.01). Of the sweet corn farmers who had a moderate level of functional health literacy, 39.53% were inclined towards a high standard of pesticide use behaviors. ( Table 6).

**Table 6.** The association between health literacy and pesticide use behaviors (n=161). Percentages represent the proportion of farmers with a particular level of HL who also have a particular standard of pesticide use behaviors. The p-value for association between health literacy and behavior, using the probability exact test. (*p<0.05,**p<0.01).

Health Literacy	Pesticide use behaviors			p-value
	Low	Moderate	High	
**Cognitive skills**				
- Low	36.36%	32.95%	30.68%	0.134
- Moderate	46.43%	25%	28.57%	
- High	11.76%	47.06%	41.18%	
**Access**				
- Low	37.50%	21.88%	40.63%	0.053
- Moderate	35.20%	35.20%	29.60%	
- High	100.0%	0%	0%	
**Communication skills**				
- Low	37.18%	31.41%	31.41%	0.226
- Moderate	0%	66.67%	33.33%	
- High	100%	0%	0%	
**Self-management**				0.006 **
- Low	0	0	0	
- Moderate	14.29%	0%	85.71%	
- High	38.31%	33.12%	28.57%	
**Media literacy**				0.053
- Low	35.00%	30.00%	35.00%	
- Moderate	37.14%	42.86%	20.00%	
- High	83.33%	0%	16.67%	
**Decision-making skills**				< 0.001 **
- Low	56.72%	28.36%	14.93%	
- Moderate	26.09%	33.33%	40.58%	
- High	16.00%	36.00%	48.00%	
**Overall of HL**				0.020*
- Low	80.00%	10.00%	10.00%	
- Moderate	33.33%	33.33%	33.33%	
- High	75.00%	25.00%	0%	
**Functional literacy**				< 0.008 **
- Low	100.00%	0%	0%	
- Moderate	25.58%	34.88%	39.53%	
- High	49.32%	28.77%	21.92%	
**Interactive literacy**				0.501
- Low	37.50%	25.00%	37.50%	
- Moderate	36.17%	32.62%	31.21%	
- High	75.00%	25.00%	0%	
**Critical literacy**				0.286
- Low	44.19%	29.07%	26.74%	
- Moderate	28.77%	35.62%	35.62%	
- High	50.00%	0%	50.00%	

## Discussion

Findings of patterns of pesticide use, health literacy and pesticide use behaviors suggest that it is necessary to reduce the harmful effects of pesticide use. Findings included (1) the majority of sweet corn farmers did not follow the current recommended dose or number of sprays, (2) there are many pesticides used in the planting of sweet corn, (3) pesticides used in growing sweet corn have moderate toxicity and (4) the pesticide use behaviors during all phases of planting are unsafe ^17^. However, assessing the risk of exposure to pesticides for humans is not easy because of differences in the duration and levels of exposure to the substance ^2, 6^. In addition, the level of HL of the farmers was moderate. Based on these findings, pesticide use by these farmers may affect environmental health ^8, 9^ and lead to the pollution of soil and water (surface and groundwater) and diseases of humans ^17^.

Therefore, training sweet corn farmers to reduce the use of pesticides ^18, 19^ through an intervention program may be necessary to improve farmer safety. These findings are also consistent with previous studies ^3, 20^ regarding health literacy (HL) and pesticide use behaviors. When the sweet corn farmers have knowledge of the pesticide being used, they may have more appropriate pesticide use behaviors. This leads to the suggestion of developing guidelines for appropriate pesticide use for each plant, following a study of the types of pesticides used for each species.

Developing HL of the risks of pesticide use requires effective communication in order to lead to an improvement in pesticide use by sweet corn farmers, which could lead to the improvement in health in this century ^21^. There are a number of ways to reduce the harmful effects of pesticide use. (1) Education for farmers about how to use PPE ^19, 22, 23^ with a focus on apron, mask and wide-brimmed hat use because the relative absorption rate of pesticides is highest in the genital area, ear canal, forehead and scalp (11.8% 5.4% 4.2% and 3.7%) ^24^. Moreover, product labels should be developed that are easy to understand. (2) Promote the use of PPE while mixing, loading and applying any pesticides, especially aprons and goggles or face shields. (3) Behavior such as eating, drinking water or alcohol and smoking should not be undertaken in areas where spraying of pesticides is necessary because the body can absorb the pesticides. (4) Farmers should take a bath at the farm to avoid contaminating family or close friends ^25^. However, farmers cannot bathe in the immediate area where they use pesticides. There are two ways to do this: use a water jug for bathing or change clothes immediately before returning home, separating the pesticide-contaminated clothing from other clothes. (5) Sweet corn planting farmers use up to nine pesticides, seven of which belong to toxicity class II according to the WHO, including 2-4D, glyphosate and atrazine that likely cause cancer ^14^. Sweet corn farmers should be careful with pesticide use, should have access to information regarding pesticide toxicity and should be encouraged to switch to organic farming methods. (6) The harm reduction principle should be applied to promote a reduction in the number of pesticides used, reduce the use of especially toxic pesticides and promote the use of the recommended pesticide concentrations. This would help to protect sweet corn farmers, others indirectly exposed to the pesticides, consumers and the environment. However, promoting the use of organic farming might be rejected by farmers as some sweet corn farmers prefer to use unsuitable pesticides in order to ensure yield and quality ^26^.

### Strengths and limitations of the study

This study was successful in collecting data with a high response rate of 82.98%. The findings of the present study can also be applied to other similar contexts. The authors suggest further qualitative study on the beliefs of farmers regarding pesticide use and organic farming as well as the study of pesticide risk reduction programs. The health literacy questionnaire was based on previously published work, pre-tested, pilot tested and edited to ensure accurate translation, coherence and relevance. However, patterns of pesticide use may be better measured using open-ended questions, where farmers are asked to provide information freely without being influenced.

## Conclusions

 The present study found that the health literacy of pesticide use in sweet corn farmers was at a moderate level and pesticide use behaviors were at a low level, with a tendency towards a high level of pesticide use behaviors. However, farmers had a moderate level of pesticide use behaviors at the ‘after pesticide application’ phase. This baseline study gives an insight into the range of pesticides used in the management of sweet corn pests and diseases in Thailand and found that 9 pesticides were used. The pesticides used in growing sweet corn have moderate toxicity. The major factors relating to pesticide use behaviors were the self-management and decision-making skills dimensions (p < 0.01). The lack of health literacy regarding pesticide calls for training for sweet corn farmers. To reduce the dependence on pesticides it is important to promote health literacy, especially in the cognitive skills, media skills and decision-making skills.

## Data availability

### Underlying data

Open Science Framework: The association between health literacy and pesticide use behaviors among sweet corn farmers in the Pak Chong district of Thailand: a cross-sectional study. https://dx.doi.org/10.17605/OSF.IO/KJ5M3 ^27^

This project contains the following underlying data:

- Data_Theerachai.csv (demographic and questionnaire response data)
- Data Dictionary_Theerachari.csv (data dictionary for Data_Theerachai.csv)
- Correct Responses_Theerachai.csv (scoring system for questionnaire responses in Data_Theerachai.csv)

### Extended data

Open Science Framework: The association between health literacy and pesticide use behaviors among sweet corn farmers in the Pak Chong district of Thailand: a cross-sectional study. https://doi.org/10.17605/OSF.IO/KJ5M3

This project contains the following extended data:

- QN-page1 - QN-page8 (questionnaire)

Data are available under the terms of access to the dataset requires registration, and is granted to those that wish to use the data for legitimate research purposes. A guide for how to apply for dataset access is available at: https://doi.org/10.17605/OSF.IO/KJ5M3 ^27^

Data are available under the terms of the Creative Commons Attribution 4.0 International license (CC-BY 4.0)
