# Supplementary material for: The association between health literacy and pesticide use behaviors among sweet corn farmers in the Pak Chong district of Thailand: a cross-sectional study
Source: F1000Res. 2019 May 15;8:448. Originally published 2019 Apr 11. [Version 2] doi: 10.12688/f1000research.18398.2 (PMC6743251; doi:10.12688/f1000research.18398.2)
Supplement: Peer review history

# Peer review: Reviewer response for version 2

**DOI:** 10.5256/f1000research.20773.r52608

**Published:** 2019-09-11

**Authors:** Archin Songthap

**Affiliations:** 1 Department of Community Health, Faculty of Public Health, Naresuan University, Phitsanulok, Thailand

**Page 1: Abstract:**

1. In methods, the authors should add the study design, sampling technique and statistics used.
2. Objectives should be: to determine patterns of pesticide use, health literacy, pesticide use behaviors, and the association between health literacy and pesticide use behaviors among sweet corn farmers in Pak Chong District, Thailand.
3. The conclusion from the findings on HL should promote focusing on the significant factors to HL including cognitive skills.

**Page 3: Introduction:**

1. In paragraph 2, please show the statistics of the increasing pesticide use among Thai farmers to see the trend.
2. As the authors mentioned about the effect of pesticide use to human health, please give an example of the screening results of the toxins prevalence in Thai farmer blood stream from a survey.

**Page 4: Methods: Statistics:**

1. Levels of health literacy can be explained by descriptive statistics.
2. If the authors use the chi-square test, please check the assumption since there are some expected values, less than 5, greater than 20% of the cells in self efficacy management and functional literacy. The authors should use Fisher exact text or Pearson chi –square test instead for these two factors.

**Page 7-8: Results:**

1. Please check percentage in each variables in Table 4. For example, communicable skill, the total of percentage in low, moderate, and high levels must be 100.0% in total, not 99.9 or 100.1%.
2. The results in Table 7, zero numbers in low level of self-management should be deleted for presentation or analysis if possible.   

I confirm that I have read this submission and believe that I have an appropriate level of expertise to confirm that it is of an acceptable scientific standard.

# Peer review: Reviewer response for version 2

**DOI:** 10.5256/f1000research.20773.r48535

**Published:** 2019-05-29

**Authors:** Kishor Atreya

**Affiliations:** 1 PHASE Nepal, Bhaktapur, Nepal

The authors have revised the manuscript given the initial comments. No theoretical relationship is sought for the health literacy and pesticide use behavior as suggested earlier, however. The revised version has a few minor corrections before indexing.

 Page 3 introduction section:

- Delete “The World health Organization……………..mancozeb (U)”.

Page 4 last paragraph:

- Delete “This analysis…….as R”. 

Page 5, last para:

- Change “pesticides” to “insecticides” and “are” to “were”.

A number of reference citation are required. Please cite reference to the following statement/tables:

- Page 5 - “According to the WHO standard and International Agency for Research on Cancer (IARC), glyphosate is likely carcinogenic to humans.”
- Table 3, Column: “Who recommends classification”.
- Table 3, Column: "IARC Carcinogenic to humans”.
- Most of the percentage figures are given in 2 digits after the decimal. 1 digit is suggested.

Page 8, 2 column, 1 para:

- Delete “however, promoting the use of organic farming……yield and quality” – this is contradiction to your earlier recommendation.

Conclusions Section:

- Delete p values. 

I confirm that I have read this submission and believe that I have an appropriate level of expertise to confirm that it is of an acceptable scientific standard.

# Peer review: Reviewer response for version 1

**DOI:** 10.5256/f1000research.20131.r47851

**Published:** 2019-04-30

**Authors:** Kishor Atreya

**Affiliations:** 1 PHASE Nepal, Bhaktapur, Nepal

Page 1, Abstract:

- “89.98%” - 90%. What about the remaining participant? In the method section, the total participant mentioned was 161, and in results section it is said 90%. Confusion!
- “Functional literacy” - What is functional literacy? Briefly define the working definition of health literacy and pesticide use behavior in the abstract.

Page 3, Introduction:

- “The top ten pesticides imported by Thailand…” - This is not required.

Page 3, Methods:

- “194 sweet corn farmers were identified” - What was the sampling method? Random and purposeful? Any sample size calculations done? What was the population of the sweet corn farmer?
- “The research framework was approved…” - It is better suited in the text later - under the heading “ethical statement”.
- “who could read and write” - This study intentionally excluded illiterate sweet corn farmers? Why? These farmers might be at higher risk of exposure?
- “6 dimensions” - Any reference?

Page 4, Methods:

- “36 items” - What are those 36 items? Please provide in the appendix.
- “low, moderate or high levels” - It is not clear that whether the authors demarcated these levels or derived from literature.
- “low, moderate and high standards of pesticide use behaviors” - Please provide 20 items in the appendix.
- “A score of <67 was considered to be low, a score of 67–69 was considered to be moderate and a score of >69 was considered to be high.” - Any literature published on these 3 categories? If so, please cite.
- “This analysis could also be performed using a non-proprietary software such as R.” - This is not required.

Page 4, Results, The demographic characteristics of the 161 sweet corn farmers:

- “male” – Males.
- “had been” – Have been.
- “mixing” - What is the mixing method?

Page 5, Results, Patterns of pesticide use:

- “four pesticide types” – Four types of pesticides.
- “pesticides” – Insecticides.
- “are” - Were

Page 5, Results, Health literacy (HL) of pesticide use:

- “2. pesticide application, 3. pesticide application” – What is the difference between 2 and 3?

Page 6, Discussion:

- “did not follow the current recommended dose or number of sprays” – I did not see any data on it in the result section.
- Ref 18 - This reference may be wrong here.
- Discussion is poor. Most of the discussion was done on the “consequences” of pesticide use, not on the results. The paper finds significant association between cognitive literacy and pesticide use behaviors, but not explained it in details. I would suggest having the results explained in the discussion section with some theory and recent literature.

Page 7, Table 6:

- I see a number of zero values in the cells. How did authors consider these values in the Chi-square test?

Page 8, Conclusions:

- “the self-management and decision-making skills dimensions (p < 0.01)” - This relationship should be explained in the discussion section.
- “The farmer has to find out…” - This is not required.

I confirm that I have read this submission and believe that I have an appropriate level of expertise to confirm that it is of an acceptable scientific standard, however I have significant reservations, as outlined above.
